# Willingness to pay to sustain and expand National Health Insurance services in Taiwan

**DOI:** 10.1186/1472-6963-8-261

**Published:** 2008-12-17

**Authors:** Hui-Chu Lang, Mei-Shu Lai

**Affiliations:** 1 Institute of Hospital and Health Care Administration, School of Medicine, National Yang-Ming University, Taipei, Taiwan; 2 Institute of Preventive Medicine, School of Public Health, National Taiwan University, Taipei, Taiwan

## Abstract

**Background:**

The purpose of the present study was to investigate people's willingness to pay to sustain the current National Health Insurance (NHI) program in Taiwan and to extend that program to cover long-term care services.

**Methods:**

A survey was administered to 1800 inpatients and 1800 outpatients, selected from health care facilities across all accreditation levels that were operating under the supervision of six different regional branches of Taiwan's Bureau of National Health Insurance (BNHI). We used a contingent valuation method with closed-ended questions to elicit participants' willingness to pay for continued national heath insurance and additional institutional long-term care services. We divided participants into six subgroups and asked individuals in these groups referendum-like yes-no questions about whether they were willing to pay one of six price bids: New Taiwan Dollar (NT$) 50, NT$100, NT$200, NT$300, NT$400, or NT$500. Logistic regression was used to analyze willingness to pay.

**Results:**

We found maximum willingness to pay for continued coverage by the NHI program and additional institutional long-term care services to be NT$66 and NT$137 dollars per month, respectively.

**Conclusion:**

We found that people were willing to pay more for their insurance coverage. With regard to methodology, we also found that using a contingent valuation method to elicit peoples' willingness to pay for health policy issues is valid. The results of the present referendum-like study can serve as a reference for future policy decision making.

## Background

The Taiwan National Health Insurance (NHI) program, which provides comprehensive health care with low premiums and cost sharing, has been one of Taiwan's most popular national policies for almost 12 years. In 2002, national health care expenditure accounted for up to 6.0% of Taiwan's gross domestic product (GDP), which is lower than that of most OECD countries. In a ranking of the quality of health care in advanced industrial and newly industrialized countries by The Economist magazine, Taiwan was placed second behind Sweden [1]. National surveys in Taiwan have found satisfaction rates for the NHI, at more than 70%, to be among the highest for national programs. The simultaneous increased in both premiums from 4.25% to 4.55% and medical center and regional hospital co-payment rates in September 2002 resulted in heated debate between political parties, non-government organizations and authorities.

Several polls were conducted after the increase in premiums and cost sharing. The results of the polls indicated that more than 60% of citizens wanted the NHI program to continue in preference to other changes; 11% wanted the number of items covered and premiums to decrease; and another 11% wanted the number of items covered to increase with a decrease in premiums. Only 15% had different opinions [2]. To ensure the continued development of the health insurance program, it was necessary to adjust the rates and further explore public opinion. In this case, the government had increased rates based on actuarial reports and had not offered the public any opportunity to comment on how the NHI could be sustained. Until now, except for the first poll held in Taiwan by the Second Generation NHI Citizen Forum [2], there has been no study undertaken that explores the extent to which Taiwanese citizens are willing to support the NHI program financially.

Contingent valuation methods to determine public willingness to pay (WTP) are well-established tools to estimate the benefits of safety and environmental policies [3-5]. Despite debates regarding methodological issues, the technique itself and its application to the evaluation of health care are becoming more widespread [6-8]. Most previous studies using WTP in health care have tried to determine the maximum amount people are willing to pay for disease treatment and management, new technology, and outcome evaluation of health care and health programs [9-15]. In developing countries, most studies have used contingent valuation methods to elicit societal WTP for community health insurance [16-18] and vaccination against infectious diseases [19-21].

Using WTP as an aid to set heath care priorities at regional or national levels has received increased interest in European countries. In one survey in Norway, Olsen and Donaldson[22] asked participants how much they would be willing to pay for three disparate health care programs within a fixed health care budget: a helicopter ambulance service; an increase in the provision of heart operations; and an increase in the provision of hip operations. That study demonstrated that it was feasible to use WTP in this context. Further methodological studies have been undertaken to examine the use of WTP in a broader, priority setting context under the auspices of a European Commission-funded project known as 'EuroWill' [23-25].

The main purpose of the present study was to elicit people's WTP for an extra premium to sustain the current NHI program in Taiwan. In addition, WTP to incorporate institutional long-term care into the NHI program was investigated. We wanted to determine what could be excluded from the comprehensive coverage currently provided by the NHI program in order to ensure its survival and to maintain premium rates at their present levels. We believe that if public opinion is taken into consideration, the political dispute following the increase in both insurance premiums and the copayment in 2002 could be mitigated and would not occur again. In the present study, we conducted a survey in various health care facilities to determine how much patients would be willing to pay to maintain the NHI program and how much they would be willing to pay for additional coverage for the new long-term care program. In addition, we gave participants a list of eight NHI programs and asked them which two they would be willing to cut in order to keep the NHI program viable. The WTP values were analyzed by logistic regression. We expect that the results of the present study will provide evidence to enable decision makers to identify suitable priorities and a reasonable price for the current NHI program.

## Methods

### Sampling method and sample size

The sample was generated by a two-stage probability proportional to size sampling. First, we selected a representative sample of hospitals across all accreditation levels of health care facilities and clinics around the entire island, which were operating under the supervision of six different regional branches of the Bureau of National Health Insurance (BNHI): Taipei, Northern, Central, Southern, Kaoping, and Eastern. Second, we calculated the sample size for each regional branch based on how much money each region was reimbursed by the NHI. Then, we distributed that sample size proportionally among each of the regional health care facilities based on the number of claims they submitted. In total, we interviewed 3600 participants in 297 hospitals and clinics. The survey was conducted in September 2003 by well-trained interviewers using the same guidelines. The proposal was reviewed by the Department of Health, Taiwan and monitored by the committee of the Second Generation Health Insurance. All participants provided informed verbal consent before interviews commenced.

### Research question

This study forms part of the Second Generation Health Insurance Project. A panel of 10 experts participated in the study and met regularly to discuss the study. We included socio demographic variables and contingent valuation questions into our analyses. Our contingent valuation question was of the closed-ended, dichotomous-choice format, which has been recommended by the National Oceanic and Atmospheric Administration (NOAA) panel on contingent valuation methods as the preferred format for determining WTP [26]. The expert panel believed that, using the referendum format, there is no strategic reason for the respondent to do other than answer truthfully. This format may more accurately reflect the decisions individuals make every day. The monetary values in the dichotomous-choice questions were determined by a panel of 10 researchers from diverse disciplines. A pretest pilot survey was conducted to revise any misinterpretation of the questions and to identify the bid vector that should be used in the final study. The bid vector was selected by accounting for the results of the pretest survey. A monthly premium was used as the payment method because this is the method currently used by the Taiwan NHI and is familiar to our respondents. The WTP questions started with a scenario that detailed a situation in which it was necessary to increase the premium in order to sustain the NHI program.

The scenario was explained as follows. Although the BNHI continues to improve administrative costs and efficiency, owing to the cost of advanced medical technology, ever-changing diseases, and an increase in the aged population, it is inevitable that the program will face financial challenges, especially if it continues to offer the current type of comprehensive health care coverage for all Taiwan's residents. Actually, Taiwan National Health Insurance has been facing a financial deficit since 1988, and the Reserve Fund is going to run out. Consider the comprehensive health insurance coverage you currently have. Imagine that one day the NHI program breaks down and you have to pay all your own heath care costs. This could happen. If you wish the NHI program to continue, you will have to pay increased premiums.

Following description of the scenario, respondents were reminded that if they agreed to pay the increased premium, they would not have these funds to, for example, purchase other goods.

The two WTP questions were as follows. First, under these circumstances, would you be willing to pay an extra [one of New Taiwan Dollar (NT$) 50, NT$100, NT$200, NT$300, NT$400, or NT$500] monthly to keep the NHI afloat? Second, would you be willing to pay an extra [one of NT$50, NT$100, NT$200, NT$300, NT$400, or NT$500] monthly to add institutional long-term care insurance to your health coverage? Each interviewee was given the option of paying only one price and the response was either 'yes' or 'no'. To ensure that the same number of participants responded to each bid amount, interviewers had to ask the questions in a consistent order. The average exchange rate was NT$34.575 for 1 US dollar during the study period.

In addition, participants were given a list of seven items covered by the NHI program (outpatient visit, chronic diseases, outpatient drugs, minor illnesses [e.g. flu and headache], half-day physical examination, Chinese medicine outpatient visit, dentistry outpatient visit) and asked which of these two, or nothing, they would be willing to cut in order to keep the NHI program viable without increasing their insurance rates.

### Statistical methods

The dichotomous-choice contingent valuation method is based on random utility theory, which assumes that choices are based on a comparison of the utility of the available alternatives, with the alternative providing the highest utility being the preferred choice [27]. Multiple logistic regression was used to estimate the mean WTP and to explore the influence of independent variables on the referendum data. The independent variables were selected on the basis of economic theory and relevant knowledge, which included the bid of WTP, gender, age, marital status, education, health status, income, and geographic area. It was assumed that the possibility of someone being willing to pay an extra premium for the NHI program to continue would be affected by the bid amount, gender, age, marital status, years of schooling, income, and residence. The lower the bid value, the higher the possibility that the respondent would be willing to pay. Women and older people were expected to be willing to pay less, whereas those with a higher income, higher education, and living in urban areas would be expected to be willing to pay more to sustain the NHI program. In addition, the demographic composition could affect WTP. The mean WTP was estimated as the area under the probability function. This area shows the proportion of the population that would consume the good at each price level and the associated utility for each of the price levels. All data were analyzed using the SAS system for Windows Version 8, SAS Institute Inc, Cary, NC, USA.

Using the logistic model, we can write the probability of answering 'yes' as:

(1) $$\log\frac{\text{prob}({\text{Y}}_{\text{i}}=1)}{1-\text{prob}({\text{Y}}_{\text{i}}=1)}=\alpha +\beta_{1}{\text{X}}_{1}+\beta_{2}{\text{X}}_{2}+\ldots +\beta_{3}{\text{X}}_{3}$$

where prob(Y_i _= 1) is the probability of answering 'yes'; α is the intercept; β is the coefficient of explanatory variables; and X is the explanatory variable.

## Results

### Survey results

We interviewed both outpatients and inpatients at the selected health care facilities (Additional file 1). In total, 1800 outpatients were interviewed, but responses for 162 subjects were excluded from analysis because of incomplete information (due, mostly, to these subjects giving up the interview in the middle of the survey), leaving us with an effective sample of 1638 and a response rate of 91%. We interviewed 1800 inpatients, but responses for 273 subjects were excluded from analysis because of incomplete information, leaving us with 1527 effective samples and a response rate of 85% (Table 1). In total, there were more women than men interviewed (53% vs. 47%, respectively) with 12% of the participants being younger than 20 years of age. The largest age group (30%) was made up of people between 21 and 35 years of age, followed by participants more than 65 years of age (14%). Categorizing the sample according to educational background, 10% of the participants were illiterate, 18% had some primary school education, and 30% had a college education or more. Forty-nine percent of participants reported a good current health status; only 17% of participants were unhealthy or extremely unhealthy. Others self-reported normal health status. Of the respondents, 63% had purchased commercial life insurance and 47% had jobs. With regard to regular monthly income, 20% of families earned less than NT$30,000, 20% earned NT$30,000–50,000, 17% earned NT$50,000–70,000, 11% earned NT$70,000–99,999, and 10% earned more than NT$100,000; 22% of respondents did not know their regular monthly income or chose not to answer the question.

**Table 1:** Survey hospital distribution and patients' demographical characteristics

Variables	Outpatients		Inpatients		Total	
	
	N = 1638	(%)	N = 1527	(%)	N = 3165	(%)
Sex						
Male	707	43.2	774	50.7	1481	46.8
Female	931	56.8	753	49.3	1684	53.2
Age						
age < = 20	243	15.1	138	9.3	381	12.3
20 < age < = 35	517	32.1	421	28.5	938	30.4
35 < age < = 50	426	26.4	361	24.5	787	25.5
50 < age < = 65	256	15.9	282	19.1	538	17.4
Age > 65	170	10.5	275	18.6	445	14.4
Marital status						
Unmarried	590	36.0	354	23.2	944	29.8
Married or partners	966	59.0	1057	69.2	2023	63.9
Divorced or disparate	30	1.8	38	2.5	68	2.2
Windowed	52	3.2	78	5.1	130	4.1
Educational background						
Illiterate	138	8.4	187	12.3	325	10.3
Less than primary school	293	17.9	269	17.6	562	17.7
Junior high school	160	9.8	242	15.9	402	12.7
Senior high school	449	27.4	471	30.8	920	29.1
Higher than college level	598	36.5	358	23.4	956	30.2
Current health status						
Very healthy	206	12.6	187	12.3	393	12.4
Healthy	650	39.7	509	33.3	1159	36.6
Normal	575	35.1	493	32.3	1068	33.8
Unhealthy	185	11.3	278	18.2	463	14.6
Extremely unhealthy	22	1.3	60	3.9	82	2.6
Purchase commercial life insurance						
Yes	1074	65.6	912	59.7	1986	62.7
No	564	34.4	615	40.3	1179	37.3
Employment						
No	828	50.6	846	55.4	1674	52.9
Yes	810	49.4	681	44.6	1491	47.1
Family annual regular income						
Less than NT$20,000	153	9.3	190	12.4	343	10.8
NT$20,000~NT$29,999	138	8.4	160	10.5	298	9.4
NT$30,000~NT$49,999	320	19.6	297	19.4	617	19.5
NT$50,000~NT$69,999	271	16.6	271	17.8	542	17.1
NT$70,000~NT$99,999	208	12.7	145	9.5	353	11.2
More than NT$100,000	177	10.8	153	10.0	330	10.4
Unknown	248	15.1	209	13.7	457	14.5
Not answered	123	7.5	102	6.7	225	7.1
Family monthly self-paid premiums						
Less than NT$500	62	3.8	68	4.4	130	4.1
NT$500~NT$999	111	6.8	114	7.5	225	7.1
NT$1,000~NT$1,499	178	10.9	168	11.0	346	10.9
NT$1,500~NT$1,999	164	10.0	132	8.6	296	9.4
NT$2,000~NT$2,999	243	14.8	241	15.8	484	15.3
NT$3,000~NT$3,999	147	9.0	139	9.1	286	9.0
NT$4,000~NT$4,999	44	2.7	76	5.0	120	3.8
More than NT$5,000	91	5.5	89	5.8	180	5.7
Unknown	578	35.3	476	31.2	1054	33.3
Not answered	20	1.2	24	1.6	44	1.4

Table 2 shows the participants' WTP to sustain the NHI program. Almost 60% of respondents stated they were willing to pay an extra premium of NT$50 per month so that the NHI program could continue; 44% stated they were willing to pay an extra NT$100. When participants were asked whether they would be willing to pay NT$200 or NT$300, the percentage of people who answered yes decreased to 34% and 25%, respectively. This indicates that the higher the extra cost, the less willingness there is to pay. At NT$500, almost 80% of respondents said they were opposed to the increase and only 18% said that they would be willing to pay. With regard to the additional coverage for institutional long-term care, 72% of respondents said they would be willing to pay an additional NT$50 for it, whereas 53% and 45% of respondents were willing to pay an additional NT$400 or NT$500, respectively. People were willing to pay more for the additional institutional long-term care than to continue NHI coverage.

**Table 2:** Patients' WTP to sustain NHI program and institutional long-term care

Willingness to pay amount	Outpatient samples N = 1638		Inpatient samples N = 1527		Total	
	
	Yes (%)	No (%)	Yes (%)	No (%)	Yes (%)	No (%)
1. How much extra money would you be willing to pay for NHI program per month for sustenance of NHI Health program?						
NT$50	60.3	39.7	54.8	45.2	57.6	42.4
NT$100	46.0	54.0	42.5	57.5	44.4	55.6
NT$200	34.7	65.3	32.9	67.1	33.8	66.2
NT$300	21.4	78.6	29.3	70.7	25.2	74.8
NT$400	19.7	80.3	23.8	76.2	21.7	78.3
NT$500	15.6	84.4	19.8	80.2	17.6	82.4
2. How much extra money would you be willing to pay for institutional long-term care service?						
NT$50	76.5	23.5	67.6	32.4	72.2	27.8
NT$100	71.9	28.1	66.9	33.1	69.5	30.5
NT$200	58.8	41.2	54.6	45.4	56.8	43.2
NT$300	53.1	46.9	53.5	46.5	53.3	46.7
NT$400	55.8	44.2	50.8	49.2	53.3	46.7
NT$500	46.5	53.5	42.7	57.3	44.6	55.4

Multiple logistic regression was used to explore the factors that influenced patients' WTP and to calculate their maximum WTP for continued NHI coverage, meaning the maximum extra monthly premium they were willing to pay to sustain the NHI program as it is at present (Table 3). We found a significant relationship for WTP bid, age, marital status, educational level, and household income with patients' WTP for continued coverage, but not such a strong relationship between gender, health status, and the area of residence with WTP for continued coverage. The results indicate that the higher the bid value, the fewer respondents would like to pay for continued NHI coverage. After controlling for other variables, respondents within the 51–60 year age group were more likely to pay than respondents who were in the younger than 35 years age group. Married respondents were less likely to pay than unmarried, divorced, or windowed respondents. Respondents with either a high school or college education were more likely to pay than respondents with only some elementary school education. The higher the household income, the greater the willingness to pay. Compared with the lowest household income level (≤ NT$29,999), respondents with higher household incomes were more likely to pay more for continued NHI coverage. By determining the area under the demand curve, we estimated that the average citizen was willing to pay an extra NT$65.8 per month for continued NHI coverage.

**Table 3:** Citizens' willingness to pay for sustenance NHI program

Parameter	β	S.E.	P
Intercept	-0.3521	0.2198	0.1093
Bid value	-0.0041	0.0003	<.0001
Sex (reference: female)			
Male	0.0070	0.0816	0.9313
Age (reference: age < = 35)			
35 < age < = 50	0.1472	0.1176	0.2106
50 < age < = 65	0.3615	0.1406	0.0101
age > 65	0.2738	0.1343	0.0415
Marital status			
Married	-0.2162	0.1002	0.0309
Education (reference: elementary school or lower)			
High school	0.253	0.1089	0.0201
College or graduate	0.3989	0.1183	0.0007
Health	-0.0195	0.0445	0.6619
Household income (reference: < = 29,999)			
30,000–69,999	0.4264	0.1207	0.0004
70,000–99,999	0.8951	0.1557	<.0001
> = 100,000	0.9751	0.158	<.0001
Unknown	0.5653	0.131	<.0001
Geographic area (reference: Taipei region)			
North region	0.0212	0.1289	0.8691
Central region	-0.2195	0.1192	0.0656
South region	0.028	0.1318	0.8321
KaoPin region	0.1922	0.1225	0.1167
East region	0.3517	0.2417	0.1456

N = 3089

-2Log L = 3615.336

Likelihood ratio test

Chi-square= 335.2738 df = 18 p < 0.0001

In the present study, we found that WTP bid, marital status, education, income, and residential location were all significantly associated with WTP for additional institutional long-term care as part of the NHI program (Table 4). This indicates that the higher the bid, the fewer respondents would be willing to pay. Married respondents were less likely to pay than unmarried, divorced, and windowed respondents. Respondents with a high school or college education were more likely to pay, respectively, than respondents with only some elementary school education. People on higher incomes were more likely to pay than those on the lowest income levels for additional institutional long-term care coverage. Compared with people living in the Taipei Branch area, residents in the Northern Branch area were significantly less likely to be willing to pay for this type of coverage. We estimated the average citizen was willing to pay an extra NT$136.5 each month to have institutional long-term care covered by the NHI program.

**Table 4:** Citizens' willingness to pay for institutional long-term care

Parameter	β	S.E.	p
Intercept	0.7394	0.2075	0.0004
Bid value	-0.0030	0.0002	<.0001
Sex (reference: female)			
Male	-0.1431	0.0775	0.065
Age (reference: age < = 35)			
35 < age < = 50	-0.1075	0.1114	0.3344
50 < age < = 65	-0.2377	0.133	0.0739
age > 65	-0.0248	0.1263	0.8445
Marital status			
Married	-0.2027	0.0955	0.0337
Education (reference: elementary school or lower)			
High school	0.236	0.1003	0.0187
College or graduate	0.3953	0.1122	0.0004
Health	0.019	0.0423	0.6535
Household income (reference: < = 29,999)			
30,000–69,999	0.4104	0.1081	0.0001
70,000–99,999	0.7048	0.1501	<.0001
> = 100,000	0.9109	0.157	<.0001
Unknown	0.4714	0.1196	<.0001
Geographic area (reference: Taipei region)			
North region	-0.8425	0.1226	<.0001
Central region	-0.22	0.1105	0.0466
South region	0.1527	0.1269	0.2288
KaoPin region	0.0313	0.1192	0.793
East region	0.0814	0.234	0.7281

N = 3089

-2Log L = 3909.394

Likelihood ratio test

Chi-square = 276.2085 df = 18 p < 0.0001

Paying more is one way of ensuring the solvency of the NHI program. Another way would be to cut some services. Therefore, in addition to asking participants about their willingness to pay more, we also presented them with a list of seven NHI items, plus the choice of 'nothing', that they may consider cutting in order to make continued NHI coverage viable. Respondents were asked to consider which two items they would cut from the list they were given to keep the NHI financially solvent. As indicated in Table 5, 33% of respondents chose to exclude minor illnesses, such as flu, headache, running nose, and skin illness, from NHI coverage, followed by half-day physical examinations, Chinese medicine, and outpatient drugs. The items least likely to be excluded by respondents were coverage for dental work, chronic disease, and all outpatient services.

**Table 5:** Given no increase in premiums, the priority of excluding service items in order to sustain NHI programs

First priority	Outpatient	Inpatient	Total
Items	N	N	N (%)
Outpatient expenditure	102	69	171 (5.5)
Chronic diseases	97	75	172 (5.6)
Outpatient drugs	126	124	250 (8.1)
Minor illnesses(flu & headache)	487	515	1002 (32.4)
Half-day physical examination	377	261	638 (20.7)
Chinese medicine outpatient	211	208	419 (13.6)
Dentistry outpatient	113	130	243 (7.9)
Nothing	100	93	193 (6.2)

Total	1613	1475	3088

## Discussion

The contingent valuation method has become the most commonly used method of non-market valuation to estimate the benefits of health goods and services. Owing to its hypothetical nature, concerns have been raised regarding the validity and reliability of contingent valuation studies. Mitchell and Carson [5] and Bateman et al. [28] stressed that the key scenario must be understandable, meaningful, and plausible. In the present study, our respondents had been in the system for more than 10 years and were familiar with the commodity to be valued. They had had prior experience evaluating and making choices regarding the consumption level of the commodity, thus increasing the likelihood that they had formulated a value for the commodity.

In the present study, we found that, on average, respondents were willing to pay an extra NT$65.8 (95% CI 64.8–66.8) each month to maintain the current NHI program and NT$136.5 (95% CI 133.8–139.2) to make institutional long-term care part of the coverage. In 2002, there was an average of 20,490,000 Taiwanese insured. If each of these people was willing to pay an extra NT$66 per month to maintain the current NHI program, then the NHI could collect an extra $NT16 billion. That respondents were willing to pay more, on average (NT$137 per month), for including institutional long-term care. The proportion of people aged 65 years and older in Taiwan has increased from 2.5% in 1951 to 9.2% in 2003 and is expected to reach 21.7% by 2036[29,30]. This has brought about an increase in the demand for long-term care for disabled and chronically ill older people in Taiwan [31]. The lack of NHI coverage for institutional long-term care adds greatly to the cost of care for these patients. In addition, because long-term care is not covered by the NHI, services are not standardized and the quality of care becomes an issue [32]. Therefore, more people in Taiwan are beginning to see the need for institutional long-term care for the elderly.

Regression analysis can be used to test the internal validity of the WTP questions. In the present study, multiple regression analysis showed household income to be a significant factor determining how much people were willing to pay for NHI and institutional long-term care services. The higher the income, the greater the likelihood that people would be willing to pay, confirming economic theory and other literature that assert that the ability to pay and WTP are closely related. As has been reported by other studies [33,34], Dong et al. [18] found that household WTP was much less than individual WTP multiplied by household size. They suspected that the heads of households did not give as much weight to the opinions of other household members as they did to their own regarding WTP. Dong et al. [18] suggested that this should be taken into consideration when setting the insurance premium. The present study only asked individuals of their willingness to pay and did not ask for household WTP. Hence, care should be taken if the data of the present study are to be used for policy making.

Marital status and education are other significant factors influencing people's WTP for continued NHI coverage and the addition of institutional long-term care to the current program. This is consistent with results of most previous studies. However, a significant geographical difference was found regarding WTP for additional institutional long-term care. Residents in the Northern Branch area were not willing to pay for additional institutional long-term care. Residents in the Southern, KaoPing, and Eastern Branch areas had a higher demand for institutional long-term care compared with residents in the Taipei area, but the difference was not significant. These results indicate the need for government policy regarding institutional long-term care and resource allocation.

Furthermore, when participants were asked which of the seven programs (or 'nothing') they would cut to keep the NHI program afloat if, hypothetically, no increase in premiums was possible, the three items most likely to be excluded were coverage for the treatment of minor diseases (32.7%), half-day physical examination (21%), and Chinese medicine (13.6%). These results may provide important suggestions for those responsible for making future decisions regarding health policy when prioritizing the value of these services. EuroWill, a European Commission-funded project, used the WTP method as a tool to measure the strength of preferences for alternative health care priorities of people in six European countries [24]. The results of the present study provide important information that could enable a similar study to be conducted in Taiwan in the near future to enable informed health policy decision making.

There are some methodological issues regarding the dichotomous, closed-ended contingent valuation method that need to be examined. With the dichotomous-choice format, in the present study respondents were asked to comment on their WTP for only one bid amount; hence, the WTP value is sensitive to the bid amounts included in the study and the number of individuals allocated to each bid amount [35]. To overcome any potential bias, we decided on the bid amounts using an expert focus group and pilot surveys; in addition, we assigned bid amounts to respondents sequentially to ensure that each bid had the same sample size. Another concern regarding the methodology is that the literature has shown that the mean WTP is much higher with the dichotomous-choice format and that there is a high proportion of 'yes' answers to the highest dichotomous-choice bid [36]. If provide a follow up question that ask respondents if they are 'yes, definitely', 'yes, probably', 'I am not sure', 'no, probably not' and 'no, definitely' and choose only whose who answer 'yes, definitely' will be helpful to limit the 'yes-saying' problem [9].

Finally, we acknowledge that our results are based on a representative sample of patients and not the general community; this may result in some kind of bias. Although the present study obtained reasonable results, the findings with respect to WTP may be overestimated and should be interpreted with caution.

## Conclusion

Although the simultaneous increase in NHI premiums and copayments caused considerable controversy, the results of the present study indicate that as long as the amount by which these rates increase is within an acceptable range, people would choose to support the continuous development of the NHI program. Even though they may complain, they would not be opposed to the implementation of higher rates. In addition, the results showed that the inclusion of institutional long-term care as part of the NHI program is an important issue. Under the current compulsory NHI program, the evolution of the program has a considerable impact on citizens' rights. The present study of WTP using a survey protocol has provided valid results, such that WTP survey for the major public interest issues could be used as an alternative decision making mechanism in addition to the prevalent elite policy making.

## Abbreviations

NHI: National Health Insurance; GDP: Gross domestic product; OECD: Organization for Economic Co-operation and Development; WTP: Willingness to pay; LTC: Long-term care.

## Competing interests

The authors declare that they have no competing interests.

## Authors' contributions

HCL and MSL both conceived of the idea and designed for this study. HCL analyzed the data and drafted the first manuscript. Both revised the manuscript and approved the final manuscript.

## Pre-publication history

The pre-publication history for this paper can be accessed here:



## Supplementary Material

Additional File 1 **The distribution of samples on geographical area and health care facilities.** The data provide the distribution of samples on geographical area and health care facilities. Click here for file

## References

[B1] EIU (2000). Health Industry: Healthcare International. The Economist Intelligence Unit London.

[B2] Huang D (2002). Citizen Forum on NHI. Taipei, Taiwan.

[B3] Jones-Lee MW (1989). The Value of Safety and Physical Risk.

[B4] Hanemann W (1994). Valuing the environment through contingent valuation. Journal of Economic Perspectives.

[B5] Mitchell RC, Carson RT (1989). Using Surveys to Value Public Goods: The Contingent Valuation Method.

[B6] Olsen JA, Smith RD (2001). Theory versus practice: a review of 'willingness-to-pay' in health and health care. Health Econ.

[B7] Diener A, O'Brien B, Gafni A (1998). Health care contingent valuation studies: a review and classification of the literature. Health Econ.

[B8] Klose T (1999). The contingent valuation method in health care. Health Policy.

[B9] Yasunaga H, Ide H, Imamura T, Ohe K (2006). The measurement of willingness to pay for mass cancer screening with whole-body PET (positron emission tomography). Ann Nucl Med.

[B10] Leighl NB, Tsao WS, Zawisza DL, Nematollahi M, Shepherd FA (2006). A willingness-to-pay study of oral epidermal growth factor tyrosine kinase inhibitors in advanced non-small cell lung cancer. Lung Cancer.

[B11] Bergmo TS, Wangberg SC (2007). Patients' willingness to pay for electronic communication with their general practitioner. Eur J Health Econ.

[B12] Sadri H, MacKeigan LD, Leiter LA, Einarson TR (2005). Willingness to pay for inhaled insulin: a contingent valuation approach. Pharmacoeconomics.

[B13] Lang HC (2005). Economic grand rounds: patients' and caregivers' willingness to pay for a cure for schizophrenia in Taiwan. Psychiatr Serv.

[B14] De Ridder A, De Graeve D Order bias in estimates of willingness to pay for drugs to treat attention-deficit/hyperactivity disorder. Eur J Health Econ.

[B15] Bishai DM, Lang HC (2000). The willingness to pay for wait reduction: the disutility of queues for cataract surgery in Canada, Denmark, and Spain. J Health Econ.

[B16] Mathiyazhagan K (1998). Willingness to pay for rural health insurance through community participation in India. Int J Health Plann Manage.

[B17] Asenso-Okyere WK, Osei-Akoto I, Anum A, Appiah EN (1997). Willingness to pay for health insurance in a developing economy. A pilot study of the informal sector of Ghana using contingent valuation. Health Policy.

[B18] Dong H, Kouyate B, Cairns J, Mugisha F, Sauerborn R (2003). Willingness-to-pay for community-based insurance in Burkina Faso. Health Econ.

[B19] Islam Z, Maskery B, Nyamete A, Horowitz MS, Yunus M, Whittington D (2008). Private demand for cholera vaccines in rural Matlab, Bangladesh. Health Policy.

[B20] Do GC, Whittington D, Le TK, Utomo N, Nguyen TH, Poulos C, Dang TD, Kim D, Nyamete A, Acosta C (2006). Household demand for typhoid fever vaccines in Hue, Vietnam. Health Policy Plan.

[B21] Whittington D, Matsui-Santana O, Freiberger JJ, van Houtven G, Pattanayak S (2002). Private demand for a HIV/AIDS vaccine: evidence from Guadalajara, Mexico. Vaccine.

[B22] Olsen JA, Donaldson C (1998). Helicopters, hearts and hips: using willingness to pay to set priorities for public sector health care programmes. Soc Sci Med.

[B23] Shackley P, Donaldson C (2002). Should we use willingness to pay to elicit community preferences for health care? New evidence from using a 'marginal' approach. J Health Econ.

[B24] Luchini S, Protiere C, Moatti JP (2003). Eliciting several willingness to pay in a single contingent valuation survey: application to health care. Health Econ.

[B25] Olsen JA, Donaldson C, Shackley P (2005). Implicit versus explicit ranking: on inferring ordinal preferences for health care programmes based on differences in willingness-to-pay. J Health Econ.

[B26] Arrow K, Solow R, Portney P, Leamer E, Radner R, Schuman H (1993). Report of the National Oceanic and Atmospheric Administration Panel on Contingent valuation. Fed Reg.

[B27] Hanemann W (1984). Welfare evaluations in contingent valuation experiments with discrete responses. Am J Agric Econ.

[B28] Bateman IJ, Carson RT, Day B, Hanemann M, Hanley N, Hett T, Jones-Lee M, Loomes G, Mourato S, Ozdemiroglu E (2002). Economic Valuation with Stated Preference Techniques.

[B29] Department of Domestic affairs (2004). Monthly Bulletin of Interior Statistics, Taiwan and Fuchien Area, R.O.C.

[B30] Department of Domestic affairs (1996). Survey of Elderly People Status in Year 1996.

[B31] Wu SZ, Chiou CJ (1997). The exploration of related factors of nursing care problems in home health care patients. J Nurs Res.

[B32] Chiu L, Tang KY, Lium YH, Shyu WC, Chang TP (1999). Cost comparisons between family-based care and nursing home care for dementia. Journal of Advanced Nursing.

[B33] Chestnut LG, Keller LR, Lambert WE, Rowe RD (1996). Measuring heart patients' willingness to pay for changes in angina symptoms. Med Decis Making.

[B34] Asgary A, Willis K, Taghvaei AA, Rafeian M (2004). Estimating rural households' willingness to pay for health insurance. Eur J Health Econ.

[B35] Cooper J, Loomis JB (1992). Sensitivity of willingness-to-pay estimates to bid design in dichotomous choice contingent valuation models. Land Economics.

[B36] Ryan M, Scott DA, Donaldson C (2004). Valuing health care using willingness to pay: a comparison of the payment card and dichotomous choice methods. J Health Econ.

