# Supplementary material for: Willingness to pay to sustain and expand National Health Insurance services in Taiwan
Source: BMC Health Serv Res. 2008 Dec 17;8:261. doi: 10.1186/1472-6963-8-261 (PMC2635366; doi:10.1186/1472-6963-8-261)
Supplement: Additional File 1 — The distribution of samples on geographical area and health care facilities. The data provide the distribution of samples on geographical area and health care facilities. [file 1472-6963-8-261-S1.doc]

Additional file 1: The distribution of samples on geographical area and health care facilities

| Variables | | Outpatients | | | Inpatients | | Total | |
| --- | --- | --- | --- | --- | --- | --- | --- | --- |
|  | | N=1638 | | (%) | N=1527 | (%) | N=3165 | (%) |
| Geographical areas | | | | | | | | |
|  | Taipei region | | 546 | 33.3 | 468 | 30.7 | 1014 | 32.0 |
|  | Northern region | | 238 | 14.5 | 238 | 15.6 | 476 | 15.1 |
|  | Central region | | 337 | 20.6 | 287 | 18.8 | 624 | 19.7 |
|  | Southern region | | 240 | 14.7 | 191 | 12.5 | 431 | 13.6 |
|  | Kaoping region | | 239 | 14.6 | 286 | 18.7 | 525 | 16.6 |
|  | Eastern region | | 38 | 2.3 | 57 | 3.7 | 95 | 3.0 |
| Hospital levels | | | | | | | | |
|  | Medical center | | 343 | 20.9 | 761 | 49.8 | 1104 | 34.9 |
|  | Regional hospital | | 314 | 19.2 | 547 | 35.8 | 861 | 27.2 |
|  | District hospital | | 225 | 13.7 | 219 | 14.4 | 444 | 14.0 |
|  | Primary clinics | | 756 | 46.2 | 0 | 0.0 | 756 | 23.9 |
| Ownership | | | | | | | | |
|  | Public | | 277 | 16.9 | 545 | 35.7 | 822 | 26.0 |
|  | Private | | 1054 | 64.4 | 332 | 21.7 | 1386 | 43.8 |
|  | Non-profit | | 307 | 18.7 | 650 | 42.6 | 957 | 30.2 |
